# Rural-specific identity and associations with lifestyle behaviors and well-being among rural cancer survivors

**DOI:** 10.1111/jrh.12835

**Published:** 2024-03-31

**Authors:** Kristina L. Tatum, Bonny B. Morris, Trevin E. Glasgow, Sam Mool (Julie) Lee, D. Jeremy Barsell, Kendall Fugate-Laus, Bernard F. Fuemmeler

**Affiliations:** 1 Department of Pediatrics, Virginia Commonwealth University, Richmond, Virginia, USA; 2 American Cancer Society, Charlotte, North Carolina, USA; 3 Department of Public Health Sciences, University of Virginia, Charlottesville, Virginia, USA; 4 School of Medicine, Virginia Commonwealth University, Richmond, Virginia, USA; 5 Department of Family Medicine and Population Health, Virginia Commonwealth University, Richmond, Virginia, USA; 6 Department of Psychology, Virginia Commonwealth University, Richmond, Virginia, USA; 7 Massey Comprehensive Cancer Center, Virginia Commonwealth University, Richmond, Virginia, USA

**Keywords:** cancer fatalism, cancer survivors, lifestyle behaviors, rural health, rural identity

## Abstract

**Background:**

Disparities in rural cancer survivors’ health outcomes are well-documented, yet the role of sociocultural aspects of rurality, such as rural identity, attitudes toward rurality, and social standing on health beliefs and behaviors remain unclear. This study aimed to address these gaps.

**Methods:**

Rural cancer survivors (*N* = 188) completed a mailed/online survey. Regression analyses identified relationships among rural identity, negative attitudes toward rurality, and social standing with health outcomes, quality of life, cancer fatalism, and cancer information overload.

**Results:**

Higher rural identity was associated with believing everything causes cancer (OR = 1.58, *p* = 0.048), believing “there’s not much you can do to lower your chances of getting cancer” (OR= 2.22, *p* = 0.002), and higher odds of being overloaded with cancer information (OR = 2.05, *p* = 0.008). Negative attitudes toward rurality was linked with higher levels of perceived stress (*B* = 0.83, *p* = 0.001), and chronic pain (OR = 1.47, *p* = 0.039). Higher subjective social status was associated with perceived social support (*B* = 0.09, *p* = 0.016), better overall health (*B* = 0.13, *p* < 0.001), lower levels of perceived stress (*B* = −0.38, *p* = 0.007), and chronic pain (OR = 0.80, *p* = 0.027).

**Conclusion:**

Sociocultural factors of rurality were associated with indicators of quality of life, cancer fatalism, and information overload. Further exploration of the underlying mechanisms that drive these associations can help improve intervention targets for rural cancer survivors.

## INTRODUCTION

Improved cancer treatment and early detection have led to cancer survivors living longer lives.^1^ Unfortunately, these positive advancements are not shared by all survivors. Health disparities persist among rural cancer survivors^2^ who are more likely to experience poorer health, worse social and emotional well-being, greater cancer burden, and lower 5-year survival rates compared to urban cancer survivors.^1–4^ Only 27% of rural Americans engage in at least 4 of the 5 cancer risk-reducing behaviors such as healthy sleep patterns, avoiding tobacco use, nondrinking or moderate drinking, maintaining healthy body weight, and meeting physical activity recommendations, compared to the overall US adult population (30.4%). Further, compared to urban residents, rural adults are less likely to meet dietary guidelines of ≥ 5 daily servings of fruit and vegetables,^5,6^ and are more likely to report lower quality of life, such as higher levels of stress, endorse chronic pain, and worse rating of health.^7^ Together, all of these health behaviors and conditions complicate cancer survivorship outcomes.

The reasons for rural health inequities, including poverty, lower education attainment, geographical isolation, and limited access to health care services have been widely studied,^3^ yet less attention has been given to the sociocultural aspects of rurality, such as rural identity, attitudes toward rurality, and social standing.^8,9^ Nechuta et al.^8^ and Blake et al.^9^ encourage the integration of sociocultural aspects of rurality in research to improve our understanding of the modifiable patterns of beliefs and behaviors that influence rural cancer survivors health disparities.

Rural identity can be described as the connection/attachment one may feel toward their community and community members.^10^ The examination of rural identity can be better understood through the lens of Social Identity Theory (SIT).^11^ According to SIT, people who identify with a particular group or community tend to adopt the values and norms of that particular community.^11^ Studies of SIT within the health literature consider how strongly a person identifies with their social group and the extent to which this self-identity predicts health behaviors, beliefs, or outcomes.^12,13^ In the context of rural identity, few studies have examined the relationship between “rural identity” and health beliefs and health outcomes to better understand rural/urban health disparities. In one study among rural adult cancer patients who lived in rural Appalachian Ohio examined rural identity, community identification, and Appalachia identity as predictors of health behaviors (clinical trial enrollment) and found no significant associations.^10^

In another study, Paskett et al.^14^ examined urban, rural, and Appalachian residents’ associations with social identity. Their results showed relationships between demographic characteristics (e.g., education, marital status, religion) with rural, Appalachian, and community identity. In addition, the authors emphasized the need for studies to examine the impact of rural identity on health and health behaviors.^14^ Oser and associates constructed a measurement of rural identity to help operationalize the definition of rurality in research.^15^ Indeed, A better understanding of one’s attachment with their community could help identify other means of intervening to improve rural cancer survivorship outcomes.

Rural attitudes and rural identity have been used interchangeably in the literature, yet they are distinct. While rural identity focuses on group attachment influences, rural attitudes focus on the attitudes (negative/positive) held by a person about a place (e.g., their community).^15,16^ Prior studies conducted among rural and urban residents assessed their attitudes toward rural life and found that positive images toward rurality were more commonly endorsed than negative images, particularly among rural residents.^17^ More recently, a study with rural and small-town residents, found associations between attitudes toward rurality with concepts related to community (e.g., community attachment and community satisfaction).^16^

Despite these associations and previous literature examining rural residents’ attitudes toward rurality,^18^ the effects and implications of rural survivors’ positive and negative attitudes on health outcomes remain unclear.

Another body of literature has examined social standing in rural populations. Work by Singh-Manoux and associates highlights the importance of subjective social status (the degree to which one perceives their social standing as better or worse than those in their community)^19,20^ as a predictor of health. Jackman suggests that subjective social status compared to objective social status better captures the perception one has of their social class in comparison to others with whom they identify within their group.^19^ The MacArthur Scale of Subjective Social Status assesses perceived socioeconomic status ranking compared to others in a similar group (i.e., The MacArthur Ladder).^21^ Research has recognized its utility in identifying associations of perceived social status in one’s community,^21^ in capturing health and disparities in racially and ethnically diverse populations, but is lacking in rural populations.^22^ The assessment of subjective social status in rural cancer survivors is needed ifwe are to better capture drivers of health disparities.

Following previous research on rural identity, attitudes toward rurality, and subjective social status, this study explores the associations that these sociocultural factors have in relation to indicators of worse outcomes among rural cancer survivors. Specifically, we hypothesized that sociocultural factors, such as rural identity, negative attitudes toward rurality, and subjective social status would be related to poorer health outcomes (physical activity, fruit and vegetable intake, Body Mass Index [BMI], and sleep), worse indicators of quality of life (lower social support, worse global rating of health, higher perceived stress, and chronic pain). In addition, the relationship between sociocultural factors and cancer fatalism and cancer information overload were examined. These beliefs have been shown to be greater among rural residents and predictive of worse outcomes among cancer survivors. We hypothesized that these beliefs would be related also to the set of sociocultural risk factors under examination.

## METHODS

### Participants and procedures

**FIGURE 1:**
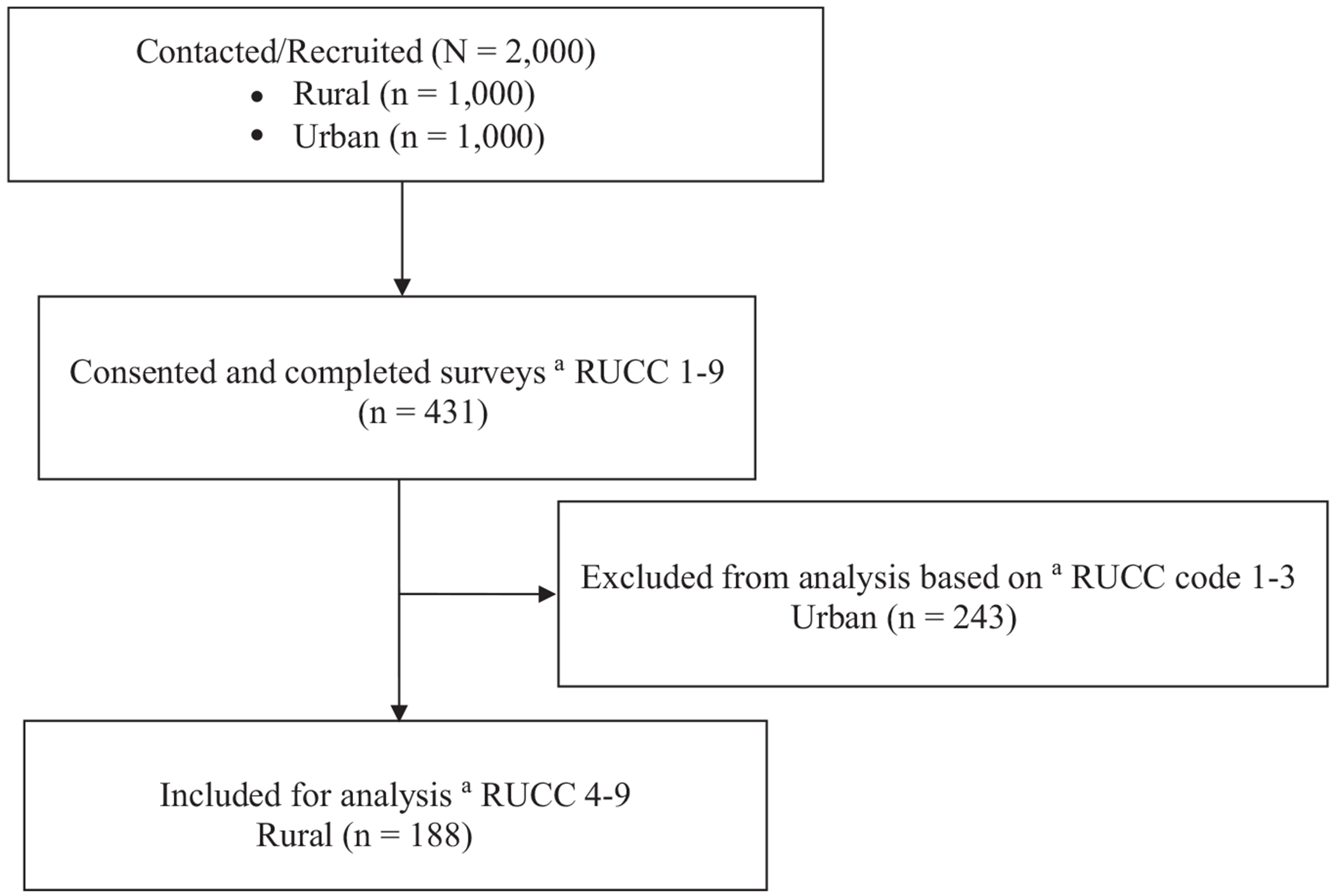
Flow diagram describing the cohort for the analyses. Abbreviations: ^a^RUCC: Rural-Urban Continuum Codes.

The current study recruited and surveyed cancer survivors residing in rural and urban residents within the catchment area of a comprehensive cancer center in the Southeastern United States.^23^ Surveys were mailed to rural 1000 and urban 1000 cancer survivors in December 2019. Cancer survivors’ rurality was determined using the United States Department of Agriculture nine levels Rural-Urban Continuum Code (RUCC).^24^ Participants with RUCC codes 1–3 were identified as living in urban areas were excluded (*n* = 431). Participants with RUCC codes 4 (population of 20,000 or more, adjacent to a metro area) to 9 (less than 2500 urban population, not adjacent to a metro area) were included (*N* = 188; Figure 1). The study was approved by the university’s Institutional Review Board. Participants had the option to complete either a mailed or online survey via Research Electronic Data Capture (REDCap).^25^

### Measures

Participants completed survey questions on social determinants of health, lifestyle behaviors, health care use, health information utilization, health history, rurality, subjective social status, and cancer screening. For the current study, health outcomes (physical activity, fruit and vegetable consumption, BMI, and sleep), quality of life (subjective social support, global rating of health, perceived stress, and chronic pain), cancer fatalism, cancer information overload, and questions related to rural identity, attitudes toward rurality, and subjective social status were analyzed. Demographic characteristics, including age, sex, race, education, and treatment status, were self-reported.

### Independent variables

#### Rural identity

Four questions assessed rural identity using a 4-point Likert scale: 1 = strongly disagree, 2 = somewhat disagree, 3 = neither agree nor disagree, 4 = somewhat agree, and 5 = strongly agree. Questions included: “Your general attitudes and opinions are similar to people who live in rural communities”; “You are typical of people who live in rural communities”; “You identify with people who live in rural communities;” and “You see yourself belonging to a rural community.”^10^ Cronbach’s alpha was acceptable (*α* = 0.83). The average of the four items was calculated to create a rural identity score. Higher scores indicated a greater association with rural place.

#### Attitudes toward rurality

Three questions assessed negative attitudes toward rurality.^18^ Questions included: “Rural people are closed-minded in their thinking,” “Rural communities provide few opportunities for the individual to get ahead in life,” and “Rural communities provide few opportunities for new experiences.” Response options were 1 = strongly disagree, 2 = somewhat disagree, 3 = neither agree nor disagree, 4 = somewhat agree, and 5 = strongly agree. Cronbach’s alpha was acceptable (*α* = 0.79). A negative attitude toward rurality score was calculated by averaging the three items, with higher values indicating more negative attitudes toward rural communities.

#### Socioeconomic position

The MacArthur Scale of Subjective Social Status was used to assess perceived social standing in relation to others in the United States and their community.^21^ Participants answered 1-item that included a picture of a ladder with 10 rungs and were asked to mark on the ladder their socioeconomic status compared to others. Scale scores range from 1 to 10, with a higher rating indicating greater perceived socioeconomic status.^21^

### Dependent variables

#### Body mass index (BMI)

Participant BMI (kg/m^2^) was calculated using self-reported height and weight. Weight categories were as follows: Overweight (BMI 25.0 to 29.9 kg/m^2^) and Obese (BMI ≥30 kg/m^2^).^26^

#### Perceived stress scale

The 4-item Perceived Stress Scale (PSS)^28^ was used to assess general stress. A sample question included, “In the last month, how often have you: felt that you were unable to control the important things in your life?” Response options were 0 = never, 1 = rarely, 2 = sometimes, 3 = very often, and 4 = always.^28^ Cronbach’s alpha was acceptable (*α* = 0.73). Perceived stress was calculated by summing the responses.

#### Perceived social support

Items assessed social and emotional support on a 6-point Likert scale (1 = never to 6 = always).^27^ Questions included: “Is there anyone you can count on to provide you with emotional support…”; “Do you have friends or family members that you can talk to about your health”; “Do you have someone to prepare your meals if you are unable to do it yourself”; “Do you have someone to take you to the doctor...”; “Do you have someone to help with your daily chores if you are sick”; and “Do you have someone to run errands if you need it?” Cronbach’s alpha was acceptable (*α* = 0.90). The total scale score was obtained by averaging participants’ responses across the 6 items. Higher scores indicated better support.

#### Chronic pain

One item was used to assess current experience with pain (“How would you rate your pain on average?”), using the validated pain numeric rating scale (NRS) ranging from 0 (“no pain”) to 10 (“worst possible pain”).^29^ Research has found a cut-off of ≥ 1 can identify pain interferences of functioning.^17^ We dichotomize responses (yes/no), with response options^29^ of 0 (“no” pain) to ≥ 1 (“yes,” pain) reported.^29^

#### Overall health

One item was used to assess current overall health status on a 5-point scale: “In general, would you say your health is...,” with response options of5 = excellent, 4 = very good, 3 = good, 2 = fair, and 1 = poor. This validated 1-item has been used in previous studies.^30,31^

#### Physical activity

The validated International Physical Activity Questionnaire self-administered short form was used to measure vigorous physical activity (VPA), moderate physical activity (MPA), and walking.^39^ Questions included: “During the last 7 days, on how many days did you do VIGOROUS physical activities like heavy lifting, digging, aerobics, or fast bicycling?”; “During the last 7 days, on how many days did you do MODERATE physical activities like carrying light loads, bicycling at a regular pace, or doubles tennis? Do not include walking,” and “During the last 7 days, how many days did you WALK for at least 10 min at a time?” The days and minutes were multiplied for VPA, MPA, and walking to obtain weekly minutes, with VPA minutes being multiplied by 2 to account for increased intensity. The weekly minutes were summed to calculate total physical activity minutes per week. Participants were categorized as meeting the recommended weekly MVPA guidelines if they engaged in physical activity ≥ 150 min a week.^32,33^

#### Sleep duration and sleep quality

Sleep duration was assessed with the following questions: “How much sleep (including naps) do you usually get on a typical weekday (e.g., workday or school day)?” and “How much sleep do you usually get on a typical weekend day (e.g., non-work or non-school day)?” Sleep quality was assessed with the following question: “In the past 7 days, how was your sleep quality?” Response options were very good, good, fair, poor, and very poor. Respondents were categorized as having at least good sleep if they reported very good or good sleep; all others were categorized as not having good sleep quality.^34,35^

#### Fruit and vegetable consumption

Fruit and vegetable intake was assessed with the following questions: “About how many cups of fruit (including 100% pure fruit juice) do you eat or drink each day?”; and “About how many cups of vegetables (including 100% pure vegetable juice) do you eat or drink each day?” Response options were ≤ 4 cups, 3 to 4 cups, 2 to 3 cups, 1 to 2 cups, ½ cup to 1 cup, ½ cup or less, and none. The number of cups of fruits and vegetables was summed together to estimate total daily fruit and vegetable consumption. This approach has been used in similar studies.^5,6^

#### Cancer fatalism and cancer information overload

Three items were used to assess fatalistic beliefs about cancer and information overload. Two items focused on cancer fatalism (“It seems like everything causes cancer” and “There’s not much you can do to lower your chances of getting cancer”) and one item asked about cancer information overload (“There are so many different recommendations about preventing cancer, it’s hard to know which ones to follow”). Response options were 1 = strongly agree, 2 = somewhat agree, 3 = somewhat disagree, and 4 = strongly disagree, with higher scores indicating higher levels of fatalistic beliefs and information overload. Items were examined separately given their low Cronbach’s alpha of 0.63.^36,37^

### Analysis strategy

In Table 1, we first calculated frequencies by RUCC 4–9. Descriptive statistics for the sample were then calculated including rural identity, negative attitudes toward rurality, subjective social status, health outcomes, quality of life, cancer fatalism, and information overload (Table 2). Correlations of independent variables were calculated, including rural identity, negative attitudes toward rurality, and subjective social status (Table 3). We conducted a series of linear and logistic regression analyses to explore associations between rurality health outcomes, quality of life, cancer fatalism, and information overload (Tables 4–6). Six regression models were conducted for each of the health outcomes: physical activity, fruit and vegetable consumption, BMI, sleep quality, weekday sleep duration, and weekend sleep duration. Four regression models were conducted for each of the indicators of quality of life: perceived social support, global rating of health, perceived stress, and chronic pain. Three logistic regression analyses were conducted for two items assessing cancer fatalism and one item assessing information overload. The following covariates were included in each regression model: age, sex, race, and education. Age was categorized into groups (19–55, 55–69, 69–95), and other demographic variables were dummy coded prior to including in the regression models. Gender was categorized into two groups: Male (reference category) or female. Race was categorized into two groups: white (reference category) and non-white race. Education was categorized into three groups: high school degree or less (reference category), some college, and college educated. Rural identity, negative attitudes toward rurality, and subjective social status were the three main outcomes included in each model. Models were fitted for each of the three main outcomes; this resulted in a total of 13 models. All analyses were conducted using SPSS, v.29.

## RESULTS

### Descriptive statistics

A total of *N* = 188 surveys were from cancer survivors in RUCC 4–9 and selected for analysis. Table 1 includes the demographic characteristics of the sample. Most of our samples were in RUCC 6 (urban population of 2500 to 19,999, adjacent to metro area). The sample mean age was 63.7 (SD = 11.7), almost a third were non-white (30%), were mostly female (64%), and a third were college graduates (34%). Of the 146 whose specific cancer diagnosis was available, the most common cancers were breast (24%), genitourinary (16%), and digestive/gastrointestinal (12%). The average time (in years) from cancer diagnosis was 3.19 (SD = 1.50). Most of the sample (73%) reported feeling that they are living comfortably/getting byon their present income, while 26% reported feeling that it is difficult/very difficult to live on their present income.

As shown in Table 2, The average rural identity score was 3.46 (SD = 0.90), while the average negative attitudes toward rurality mean was 2.98 (SD = 1.07), suggesting respondents identified moderately with rural identity and held moderate negative attitudes toward rural communities. The average reported subjective social status rating was 6.30 (SD = 2.12), indicating greater than average perceived social status rating.

Most cancer survivors (67%) met the weekly MVPA recommended guidelines of 150 min of MVPA per week. The average total daily fruit and vegetable consumption (in cups) was 2.76 (SD = 1.93). Rural cancer survivors mean weight was 29.87 (SD = 8.04), which is classified as “overweight.” Only 15% (27 cancer survivors) reported at least “good” sleep quality. The average sleep duration (in hours) on weekdays was 7.36 (SD = 1.63), with the average higher on weekends was 7.65 (SD = 1.79). Approximately 50% of respondents reported experiencing pain. The average support score was 4.55 (SD = 0.80) and the overall health rating was 2.79 (SD = 0.93). The average perceived stress score was 4.84 (SD = 3.19).

Fatalistic beliefs about cancer and information overload showed that many rural cancer survivors (63%) agreed with the statement, “It seems like everything causes cancer,” and 73% agreed with the statement, “There are so many different recommendations about preventing cancer, it’s hard to know which one to follow.” Most disagreed (64%) with the statement “There is nothing you can do to lower your chances of getting cancer.”

Table 3 presents correlations of the independent variables. Pearson’s correlation coefficient showed no significant associations.

### Multiple linear and logistic regression model results

Table 4 provides six regression models for overall health outcomes on results for physical activity, fruit and vegetable consumption, BMI, and sleep. Race was a significant predictor of higher BMI (*B* = 3.27, *p* = 0.044), and lower weekday (*B* = −0.95, *p* = 0.013) and weekend (*B* = −1.29, *p* = 0.002) for non-white cancer survivors. Furthermore, older cancer survivors (OR = 0.40, *p* = 0.032) were more likely to report at least “good” sleep quality. Total weekly physical activity and daily fruit and vegetable consumption were not associated with any of the independent variables.

Table 5 presented regression results for quality of life variables (perceived social support, global rating of health, and perceived stress). Perceived social support was significantly associated with a higher rank of social status (*B* = 0.09, *p* = 0.016). Having a college education (*B* = 0.52, *p* = 0.007) and higher social status (*B* = 0.13, *p* < 0.001) were associated with a better perceived global health rating. Older cancer survivors (*B* = −1.17, *p* = 0.005) and higher subjective social status (*B* = −0.38, *p* = 0.007) were associated with lower perceived stress. On the contrary, negative attitudes toward rurality (*B* = 0.83, *p* < 0.001) was associated with more perceived stress. Additionally, race (OR = 2.92, *p* = 0.023), negative attitudes toward rurality (OR = 1.47,*p* = 0.039), and higher subjective social status (OR = 0.80, *p* = 0.027) were significantly associated with greater pain.

Table 6 presents logistic regression (i.e., odds ratio [OR], 95%, confidence intervals, and *p*-values) three models predicting associations with cancer fatalism and cancer information overload. Higher rural identity was associated with higher odds of believing “It seems like everything causes cancer” (OR = 1.58, *p* = 0.048), “There is nothing you can do to lower your chances of getting cancer” (OR = 2.22, *p* = 0.002), and “There are so many different recommendations about preventing cancer, it’s hard to know which one to follow” (OR = 2.05, *p* = 0.008).

## DISCUSSION

Overall, our findings showed rural identity, negative attitudes toward rurality, and subjective social status differ in their associations with health outcomes (physical activity, fruit and vegetable consumption, BMI, and sleep), quality of life (perceived social support, global rating of health, perceived stress, and chronic pain), in addition to cancer fatalism, and information overload among rural survivors. Specifically, higher rural identity was associated with fatalistic beliefs about cancer and information overload. Those with negative attitudes toward rurality reported higher levels of perceived stress and were more likely to endorse chronic pain. Further, subjective social status was significantly associated with higher ratings of overall health, higher perceived social support, lower levels of perceived stress, and higher odds of reporting chronic pain.

We found rural identity was positively associated with fatalistic beliefs about cancer. Similar associations have been found in rural Appalachia Kentucky residents who endorsed the fatalistic belief that “there is nothing you can do to reduce my risk of developing colorectal cancer.” These residents were less likely to have an endoscopy.^37^ It is plausible that a higher rural identity relationship with fatalistic beliefs is attributed to having a stronger group identification with similar beliefs in their community.^38^ Moreover, we found rural identity was higher among rural cancer survivors who felt overwhelmed and frustrated by the amount of cancer-related information. Jensen and associates found that rural residents reported higher levels of fatalistic beliefs about cancer and cancer information overload compared to urban residents.^39^ The authors described the role of psychological stress and coping theory on cognitive appraisal (perception of control via avoidance of stress to cope with contextual challenges) as a potential explanation for these associations among rural cancer survivors.^40^ For example, they posit that fatalistic thinking reflects how an individual cognitively appraises a situation by “reducing, revising, avoiding” to manage stressors (e.g., lack of resources/access).^39^ A potential approach to address fatalistic thinking and information overload, is to understand factors that drive cancer fatalism, such as group identification, as well as connect rural residents with community-based organizations to enhance the quality of information delivery.^42^

Negative attitudes toward rurality was associated with worse indicators of quality of life (higher perceived stress and endorsed chronic pain). Negative attitudes toward rurality and worse psychological and physical outcomes could be accounted for by the limited availability of resources (e.g., > 1 h to treatment) in rual locations to manage health concerns.^43^ Disparities in chronic pain management in rural communities are in part due to a lack of resources and access to care. Prior findings have shown a higher prevalence of chronic pain and perceived stress among rural residents compared to non-rural residents^7,44^. More research would benefit from further understanding the relationships between negative attitudes toward rurality and rural cancer survivors’ quality of life. Identifying the role of negative attitudes toward rurality on indicators of quality of life as well as how rural residents’ attitudes are impacted by access to resources could inform intervention targets.

We found significant associations between subjective social status with levels of perceived stress, indicating higher perceived socioeconomic standing was associated with lower levels of perceived stress.^20^ It may be that the perception of social status has a greater impact on health than the actual metrics of social status.^21^ In one study with a rural population of non-cancer survivors,^45^ research on perceived social status and self-reported health and well-being demonstrated an association between lower perceived social status with poorer self-rated health. These results indicated that higher perceived social status may be associated with better health.^22^ The examination of rural cancer survivors’ perceived social status is lacking. This limits our understanding of how one’s perception of their social status impacts rural survivors’ overall health.

Lastly, other findings included associations with health outcomes. We found those who reported their race as non-white had a higher BMI, reported greater chronic pain, and had worse weekday and weekend sleep duration. Our results are similar to previous studies among rural residents and racial/ethnic minorities.^46,47^ Future research is warranted to better understand the factors that drive these associations among cancer survivors in rural areas.

### Strengths and limitations

This study was conducted with cancer survivors only in one cancer center’s catchment area, which may limit the generalizability of the findings to other US cancer survivors. The rural identity and negative attitudes toward rural measures have been used in prior studies, however, more research is needed to test the validity of these items. Additionally, since all of our measures were self-reported, the participants could have overestimated or underestimated their behaviors. Further, we recognize the limitations of our analyses of running 13 multiple regression models, which increased the probability of false-positive findings.

Despite these limitations, our study had several strengths. We had a robust sample size of rural cancer survivors (*N* = 188). While we had incomplete data on cancer diagnosis for more than 40 participants, we did not have to rely on self-reported cancer diagnoses. While most other studies have focused on geographical location as the main predictor of health among rural populations, this study uniquely focused on associations with sociocultural factors of rurality. Our results are novel, and the value of our findings is important given that this population of rural cancer survivors is largely understudied.

## CONCLUSION AND FUTURE DIRECTIONS

Our results identified associations between rural identity, negative attitudes toward rurality, and subjective social status with quality of life, cancer fatalism, and information overload among rural cancer survivors, which has been previously unexplored. Future research should extend our findings by integrating sociocultural aspects of rurality to help ascertain potential mechanisms through which group identification can either exacerbate or buffer health outcomes. Furthermore, follow-up research is needed to further investigate our significant findings including examining longitudinal associations to improve interventions targets and mitigate disparities among rural cancer survivors.

## Figures and Tables

**TABLE 1:** Demographics characteristics of the rural cancer survivors.

		(*N* = 188) *n* (%)	Mean (SD)
Rural-Urban Continuum Codes			
4	Urban pop > 20,000, adjacent to a MA	4 (2.1)	
5	Urban pop > 20,000, not adjacent to MA	0(0)	
6	Urban pop of 2500 to 19,999, adjacent to MA	86 (45.7)	
7	Urban pop of 2500–19,999, not adjacent to MA	30 (16.0)	
8	Completely rural or <2500 urban pop, adjacent to MA	38 (20.2)	
9	Completely rural or <2500 urban pop, not adjacent to MA	30 (16.0)	

Age			63.72 (11.73)

Female		180 (64.4)	

Black/other		179 (30.4)	

Education			
	High school or less	65 (36.1)	
	Some college or post high school training	54 (30.0)	
	At least college graduate	61(33.9)	

Time since cancer diagnosis (in years)			3.19 (1.50)

Type of cancer			
	Breast	31 (24.0)	
	Digestive/gastrointestinal	17 (12.0)	
	Endocrine and neuroendocrine	10 (7.0)	
	Genitourinary	12 (16.0)	
	Gynecologic	14 (10.0)	
	Head and neck	10 (7.0)	
	Heart	3 (2.0)	
	Hematologic/blood	10 (7.0)	
	Neurologic	5 (3.0)	
	Respiratory/thoracic	13 (9.0)	
	Skin	9 (6.0)	
	Unknown primary	1 (1.)	

Which one of these come closest to your own feelings about your household’s income these days?	Living comfortably on present income	69 (38.1)	
	Getting by on present income	64 (35.4)	
	Finding it difficult on present income	128 (15.5)	
	Finding it very difficult on present income	20 (11.0)	

*Notes*: Percentages were rounded to the nearest whole number and may not sum to 100.

Abbreviations: pop, population; MA, metro area; RUCC, Rural-Urban Continuum Codes.

**TABLE 2:** Descriptive statistics for the sample.

	(*N* = 188)	
	*n*(%)	Mean (SD)
^a^Rural identity		3.46 (0.90)

^a^ Negative attitudes toward rurality		2.98 (1.07)

^b^Subjective social status		6.30 (2.12)

Fatalism and information overload		

Everything causes cancer		
Disagree	67 (37.2)	
Agree	113 (62.8)	

Prevent not possible		
Disagree	115 (63.9)	
Agree	65 (36.1)	

Too many recommendations		
Disagree	49 (26.9)	
Agree	133 (73.1)	

Health and quality of life		
^c^Perceived stress (4-item scale)		4.84 (3.19)
^d^Perceived support (6-item scale)		4.55 (.802)

Chronic pain		
Yes	91 (51.1)	
No	87 (48.9)	

^e^Overall health		2.79 (.931)

BMI and health Behaviors		
BMI		29.87 (8.0)

At least “good” sleep quality (compared to only fair, poor, or very poor)	27 (14.8)	
Weekday sleep duration (in hours)		7.36 (1.63)
Weekend sleep duration (in hours)		7.65 (1.79)
Fruit and vegetable consumption		2.76 (1.93)

Percent who meet MVPA (including walking) weekly guidelines of 150 min per week	108 (67.1)	

Abbreviations: BMI, body mass index; MVPA, moderate to vigorous physical activity; RUCC, Rural-Urban Continuum Codes.

a Rated on a 1 to 5 scale where 1 is “strongly disagree” and 5 is “strongly agree.”

b Rated from 1 to 10 where 1 is lower subjective social status and 5 is higher subjective social status.

c Rated on a 0 to 4 scale where 0 is “never” and 4 is “always.”

d Rated on a 1 to 6 scale where 1 is “never” and 6 is “always.”

e Rated on a 1 to 5 scale where 1 is “poor” and 5 is “excellent.”

**TABLE 3:** Correlation for independent variables (*N* = 188).

Variables	1	2	3
1. Rural identity	.	.	.
2. Negative attitudes toward rurality	−0.105	.	.
3. Subjective social status	0.141	−0.158	.

**TABLE 4:** Six sets of regression results predicting overall health outcomes.

Independent variables	Met MVPA weekly guidelines				Fruit and vegetable intake			Body mass index		
	OR	95% CI		*p* Value	*B*	SE	*p* Value	*B*	SE	*p* Value
Age	0.52	0.26	1.06	0.072	−0.06	0.26	0.807	−1.84	1.04	0.079
^a^ Female	1.78	0.74	4.30	0.198	0.37	0.37	0.325	−0.22	1.46	0.878
^b^ Non-white race	0.40	0.15	1.06	0.065	0.52	0.41	0.210	3.27	1.60	0.044
^c^ Some college	1.03	0.34	3.11	0.965	0.70	0.45	0.127	−2.71	1.79	0.133
^c^ College educated	1.50	0.49	4.46	0.488	0.87	0.45	0.057	−0.42	1.77	0.813
Rural identity	1.12	0.69	1.83	0.645	−0.11	0.20	0.599	1.12	0.79	0.161
Negative attitudes toward rurality	1.34	0.90	2.05	0.148	−0.00	0.16	0.987	0.62	0.63	0.327
Subjective social status	1.07	0.86	1.33	0.555	0.01	0.09	0.875	0.68	0.35	0.051
Adjusted *R*^2^					*R*^2^ = −0.001, *F*(8, 120) = 0.983, *p* = 0.452			*R*^2^ = 0.069, *F*(8, 117) = 2.160, *p* = 0.036		
Nagelkerke *R*^2^	0.208									
Independent variables	Report at least “good” sleep quality				Weekday sleep duration			Weekend sleep duration		
	OR	95% CI		*p* Value	*B*	SE	*p* Value	*B*	SE	*p* Value
Age	0.40	0.17	0.93	0.032	0.46	0.24	0.061	0.01	0.25	0.975
^a^ Female	1.36	0.40	4.56	0.622	−0.57	0.33	0.082	−0.28	0.36	0.430
^b^ Non-white race	1.97	0.57	6.78	0.285	−0.95	0.38	0.013	−1.29	0.40	0.002
^c^ Some college	0.96	0.24	3.84	0.956	−0.59	0.40	0.152	−0.39	0.44	0.373
^c^ College educated	0.32	0.07	1.51	0.150	−0.52	0.40	0.195	−0.72	0.43	0.096
Rural identity	0.54	0.28	1.04	0.066	0.06	0.17	0.733	0.33	0.19	0.093
Negative attitudes toward rurality	1.42	0.82	2.41	0.196	0.08	0.14	0.572	−0.02	0.15	0.908
Subjective social status	1.04	0.80	1.34	0.778	−0.07	0.08	0.383	−0.07	0.09	0.448
Adjusted *R*^2^					*R*^2^ = 0.099, *F*(8, 96) = 2.430, *p* = 0.019			*R*^2^ = 0.106, *F*(8, 104) = 2.659, *p* = 0.011		
Nagelkerke *R*^2^	0.160									

Abbreviations: CI, confidence interval; OR, odds ratio for categorical outcomes.

a Ref: male.

b Ref: white.

c Ref: high school or less.

**TABLE 5:** Four sets of regression results predicting quality of life.

Independent variables	Perceived social support			Global rating of health			Perceived stress		
	*B*	SE	*p* Value	*B*	SE	*p* Value	*B*	SE	*p* Value
Age	−0.00	0.12	0.988	0.05	0.11	0.666	−1.17	0.41	0.005
^a^ Female	−0.30	0.15	0.057	0.13	0.15	0.397	0.95	0.58	0.104
^b^ Non-white race	−0.10	0.17	0.579	−0.04	0.17	0.814	−0.45	0.64	0.480
^c^ Some college	0.01	0.19	0.944	0.25	0.19	0.191	−0.48	0.71	0.498
^c^ College educated	0.11	0.19	0.561	0.52	0.19	0.007	−0.81	0.70	0.248
Rural identity	0.13	0.08	0.110	0.10	0.08	0.231	−0.07	0.31	0.835
Negative attitudes toward rurality	−0.05	0.07	0.425	−0.06	0.07	0.349	0.83	0.25	0.001
Subjective social status	0.09	0.04	0.016	0.13	0.04	<0.001	−0.38	0.14	0.007
Adjusted *R*^2^	*R*^2^ = 0.084, *F*(8, 118) = 2.441, *p* = 0.018			*R*^2^ = 0.190, *F*(8, 120) = 4.750, *p* < 0.001			*R*^2^ = 0.216, *F*(8, 116) =5.275, *p* < 0.001		
Independent variables		Chronic pain							
		OR			95% CI				*p* Value
Age		1.01			0.56		1.81		0.974
^a^ Female		1.46			0.64		3.34		0.374
^b^ Non-white race		2.92			0.16		7.31		0.023
^c^ Some college		1.39			0.52		3.71		0.517
^c^ College educated		0.72			0.27		1.93		0.509
Rural identity		1.27			0.81		2.00		0.304
Negative attitudes toward rurality		1.47			1.02		2.11		0.039
Subjective social status		0.80			0.66		0.98		0.027
Nagelkerke *R*^2^		0.208							

Abbreviations: CI, confidence interval; OR, odds ratio for categorical outcomes.

a Ref: male.

b Ref: white.

c Ref: high school or less.

**TABLE 6:** Three sets of regression results predicting cancer fatalism and information overload.

Independent variables	^a^ Everything causes cancer				^b^ Prevent not possible				^c^ Too many recommendation			
	OR	95% CI		*p* Value	OR	95% CI		*p* Value	OR	95% CI		*p* Value
Age	1.02	0.57	1.80	0.959	0.74	0.40	1.37	0.343	1.47	0.79	2.74	0.227
^d^ Female	0.88	0.39	2.00	0.755	0.74	0.32	1.74	0.491	0.39	0.15	1.05	0.063
^e^ Non-white race	1.63	0.63	4.22	0.310	2.42	0.96	6.10	0.060	2.22	0.76	6.48	0.145
^f^ Some college	1.20	0.43	3.37	0.719	0.72	0.26	2.00	0.528	2.03	0.64	6.43	0.229
^f^ College educated	1.03	0.38	2.81	0.944	0.80	0.29	2.22	0.670	2.18	0.72	6.65	0.169
Rural identity	1.58	1.00	2.48	0.048	2.22	1.33	3.70	0.002	2.05	1.21	3.47	0.008
Negative attitudes toward rurality	1.15	0.79	1.66	0.479	1.12	0.78	1.60	0.554	0.96	0.63	1.47	0.861
Subjective social status	0.87	0.72	1.07	0.184	0.88	0.72	1.07	0.205	0.84	0.67	1.04	0.106
Nagelkerke *R*^2^	0.074				0.185				0.160			

Abbreviations: CI, confidence interval; OR, odds ratio for categorical outcomes.

a It seems like everything causes cancer.

b There’s not much you can do to lower your chances of getting cancer.

c There are so many different recommendations about preventing cancer; it’s hard to know which ones to follow.

d Ref: male.

e Ref: white.

f Ref: high school or less.
